# Revisiting students’ foreign language learning demotivation: From concepts to themes

**DOI:** 10.3389/fpsyg.2022.1030634

**Published:** 2022-10-03

**Authors:** Lixiang Gao, Honggang Liu

**Affiliations:** ^1^Faculty of Education, Northeast Normal University, Changchun, China,; ^2^School of Foreign Languages, Soochow University, Suzhou, China

**Keywords:** demotivation, foreign language learning, demotivators, concepts, themes

## Abstract

Demotivation is a common psychological phenomenon in foreign language learning. Having a good understanding of learners’ foreign language learning demotivation is conducive to enriching the fruits of psychological research on foreign language learning theoretically and practically by exploring effective ways to improve learners’ foreign language learning motivation. Therefore, this study entails an analysis of the selected literature from 2001 to 2021 to interpret the concept of foreign language learning demotivation, illustrate the research topics from the classification of demotivators and their relationships with other psychological factors, and fully describe the research methods and participants. Future research should expand theoretical perspectives, include more participants of different grades, and adopt multiple research methods.

## Introduction

Since Chambers (1993) first explored demotivation in second language (L2) learning (Dörnyei and Ushioda, 2021), there has been an increasing interest in studying students’ learning demotivation in language education (e.g., Falout and Maruyama, 2004; Kikuchi, 2009; Kim and Seo, 2012; Hassaskhah et al., 2014; Xaypanya et al., 2017; Kim et al., 2019; Karaca and Inan, 2020; Thorner and Kikuchi, 2020; Gao et al., 2022). Some factors influencing students’ demotivation have been identified, and they may be related to the students’ own psychological changes, such as excessive learning anxiety (Xaypanya et al., 2017), reduced confidence (Falout and Maruyama, 2004), and negative language attitude (Kaivanpanah and Ghasemi, 2011). Alternatively, they may relate to the learning environment, such as boring learning material (Husniyah, 2019), challenging tasks (Lai, 2013), and disappointing exam results (Sakai and Kikuchi, 2009). To deeply explore these demotivators (factors leading to learners’ demotivation), many scholars have made great achievements in their research on foreign language learning (FLL) demotivation in various contexts in the past 30 years. Therefore, there is a need to scrutinize these studies, present their findings, and illustrate their research designs. With these ideas in mind and by analyzing the retrieved research, our review aims to answer the following questions:

1. How is FLL demotivation defined?
2. What are the major themes in the demotivation research?

Reviewing the body of research on FLL demotivation will allow for addressing these questions. Such a review can produce a summary and definition of demotivation but also allow for considering how demotivators influence students. Practically, this review will identify demotivators and explore the reasons for students’ FLL demotivation, which will be beneficial in the search for ways to remotivate students in the classroom practice. Theoretically, this review may enrich the fruits of demotivation research, highlight research gaps, and offer some implications for future studies. Specifically speaking, theoretical perspectives are suggested to expand; more participants of different grades should be included; and multiple research methods should be adopted to achieve triangulation.

## Methodology

Two separate electronic search engines (Scopus database and Web of Science) were used to retrieve international studies. Both literature searches relied on the keywords “demotivation,” “demotivating,” and “demotivator” appearing in the title, abstract, or keywords. Since a working definition of demotivation was offered in 2001 by Dörnyei, we ran our searches and limited the publication date to the period 2001 to 2021 (i.e., articles published in the past 21 years). Therefore, the first round of browsing retrieved 976 pieces of literature.

In selecting the related literature, we followed Hollier (2020) literature screening steps: deduplication, screening for relevance with titles and abstracts, and screening full texts with critical appraisal techniques. First, we restricted the domain and types of literature and concentrated on journals, books, and book chapters in the areas of social science and psychology. Then the retrieved records were exported, and by using the reference management software NoteExpress, we were able to eliminate duplicates. This yielded 325 pieces of literature. After a careful screening of titles, we found 76 potential records related to language learning (three books, four book chapters, and 69 articles), which consisted of the primary review data pool. We adhered to the following criteria (Macaro et al., 2018; Rose et al., 2021) to include and exclude literature in the final round of literature selection. The included reviewed literature needed to be empirical studies on demotivation in L2 learning or FLL. Furthermore, these works needed to be published in peer-reviewed journals. Doctoral dissertations were also included, since they were sufficiently peer-reviewed. It is worth noting that some works that were not empirical studies but were well acknowledged by peers were also included (e.g., Dörnyei, 2001; Dörnyei and Ushioda, 2021). Such records would be discarded if they were theoretical articles or reviews, since they were not conducted within the space of theory and practice (Rose et al., 2021). Master’s dissertations were excluded because they were not sufficiently peer-reviewed.

**Figure 1:**
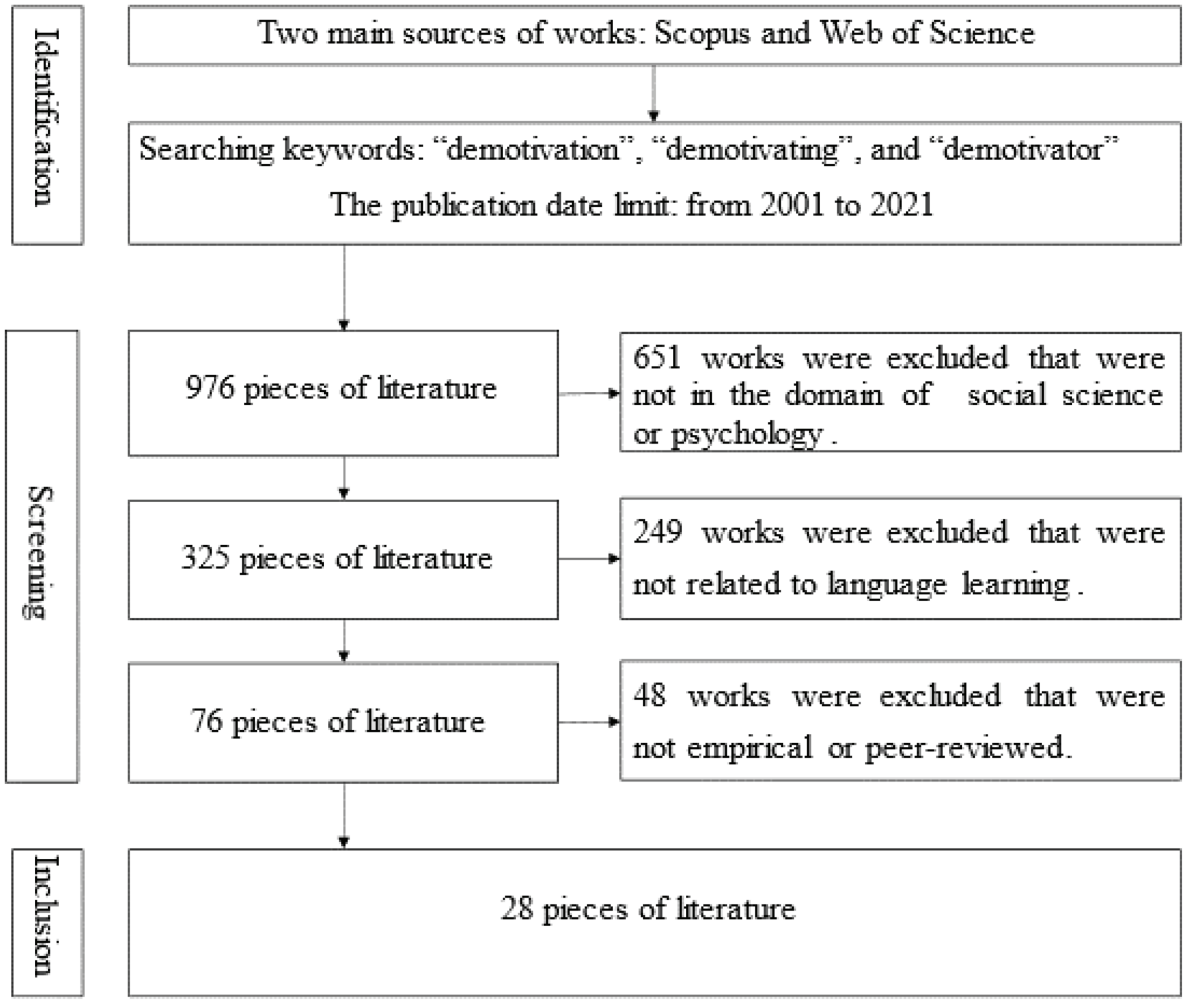
Process of literature selection.

Following the rules above, we read the abstracts and full texts of the 76 works to determine whether to include them in our final data pool. Ultimately, 48 studies were excluded. Therefore, 28 full-text studies (three books, one book chapter, and 24 articles) from the initial selection were retained and deeply explored (Figure 1): 22 reporting research on FLL demotivators in various contexts and six reporting research on the relationship between demotivators and other psychological factors, such as anxiety, resilience, and engagement.

## Findings and discussion

We two authors separately analyzed the data considering the samples, research methods, and themes. These three aspects were closely related to the research questions. We both read the full text of each work with the purpose of combing the three aspects. If there were any difficulties in dealing with data, we would re-examine the study and solve these difficulties. Finally, from the analysis and discussion of the three aspects, four topical issues emerged: definitions of demotivation, classifications of demotivators, their relationships with other psychological factors, and methodological approaches.

### Definitions of demotivation: The joint effects of factors interaction

L2 demotivation is not a complete loss but a decreased level of L2 learning motivation (Dörnyei, 2001). Until now, more research has been focused on language learning motivation, while “the other side” of motivation (Falout and Falout, 2005) - demotivation has been less researched. Meanwhile, exploring language learning demotivation is helpful to provide solutions to stimulating students’ interest in learning a foreign language. Therefore, there is a need for the exploration of language learning demotivation. But most researchers tend to explore factors influencing learners’ demotivation and ignore what demotivation is. Nevertheless, understanding its connotation will help us understand how students become demotivated. Therefore, there is an urgent need to explore its definition.

A few researchers have expounded on what demotivation is, among whom Dörnyei’s team is well acknowledged. Dörnyei (2001) was known for conducting the first in-depth exploration of the concept of demotivation. He argued that demotivation concerned a series of external forces that could weaken an individual’s behavioral intention and the motivational basis of an ongoing activity. Demotivated learners once had a strong learning motivation but lost this interest for external reasons. Dörnyei and Ushioda (2021) further developed the definition of demotivation by asserting that demotivation was a negative process in which a person’s behavioral motivation relating to intention or ongoing activities was diminished. This definition refers to demotivation as a negative process experienced by individuals instead of emphasizing the external causes of demotivation. In addition, Kikuchi (2015) argued that demotivation was a negative process that dragged learners down and diminished their motivation, including internal and external factors.

As we can see, Dörnyei (2001), Dörnyei and Ushioda (2021), and Kikuchi (2015) all emphasize factors influencing learners’ demotivation when defining it and highlight that it is a negative process that prevents learners from progressing.

From what has been achieved in the past 20 years, it is apparent that demotivation is usually referred to as a negative emotional process influenced by internal and external factors. In this process, learners themselves will suffer from some negative psychological changes and thus experience low confidence, excessive anxiety, and negative language attitude. In addition, English course, teachers, and the environment will act on learners, causing the decline of their learning motivation. However, it is noteworthy that most relevant studies emphasize the impact that a single factor has on learners’ demotivation instead of exploring the interaction among these factors. In fact, from the perspective of Bronfenbrenner (1986) ecological systems theory, as active individuals, learners will interact with elements in their surroundings at any time, and these elements are likely to have mutual effects and lead to demotivation. Therefore, demotivation is not a simple result of these factors accumulating but of their interaction and reciprocal influence. In this study, demotivation is defined as a negative process where learners’ motivation drops from a higher level to a lower level due to internal and external factors’ joint and mutual effects.

### Classifications of foreign language learning demotivators

**Figure 2:**
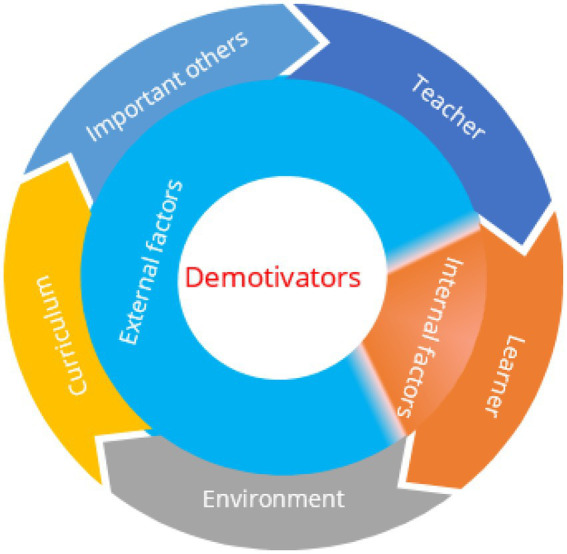
The classifications of demotivators.

A careful review of related studies on FLL demotivation shows that most studies focus on FLL demotivators. Although the results may vary, they share some similarities in their classification of demotivators. Generally speaking, demotivators in FLL mainly include internal and external factors (Figure 2). The internal factors are mainly composed of learner-related factors (Falout et al., 2009), while the external factors are mainly teacher-related, environment-related, important others-related, and curriculum-related factors (Lai, 2013).

#### Learner-related factors

Learner-related factors refer to the fact that learners’ motivation will be diminished if they experience negative learning emotions (Elahi Shirvan and Talebzadeh, 2020), such as reduced confidence, decreased interest in learning, negative language attitude, and high study anxiety (Falout et al., 2009).

First, a decline in learners’ confidence does not mean they are not confident in themselves but that they suffer a gradual loss in confidence. Learners’ confidence will be affected by various factors and will decrease from a higher to a lower level (Dörnyei, 2001). This is especially true for students with lower language proficiency. For example, Falout and Falout (2005) found that students with low proficiency tended to attribute demotivation to themselves. They believed that their learning demotivation was due to their incapacity to learn English well and failure to control their emotions. Worse still, they were likely to fall into a malignant emotional circle: blaming themselves – performing poorly – blaming themselves again – performing poorly. Second, the inherent complexity of the English language may account for students’ decreased interest (Sakai and Kikuchi, 2009). For example, there may be too many words to recite and boring grammar to understand. This will make students less active in English class and they will get tired of learning English, thus becoming demotivated. Besides, some students may not realize the importance of learning English and regard it as a challenging subject (Kaivanpanah and Ghasemi, 2011). They hold a wrong belief that English is useless to their future and there is no point in learning it. Lastly, excessive anxiety will lead to some bad results. Students will care more about others’ attitudes and will fear being laughed at if they fail to use English correctly. Trapped in the situation of being worried and making mistakes, they will tend to avoid any occasion to use English (Xaypanya et al., 2017).

#### Teacher-related factors

Teacher-related factors refer to the dark side of teachers’ teaching behaviors, professional qualities, and teaching skills (Kikuchi, 2009). Regarding their closeness to teachers, these factors can be divided into two categories: one directly related to teachers and the other indirectly related to teachers (Falout and Falout, 2005). The former includes teachers’ basic teaching skills, teachers’ sense of responsibility, teachers’ personality, the teacher–student relationship, and teaching methods. Specifically, teachers’ poor teaching skills are reflected in non-standard pronunciation, incomprehensible explanations, and so on (Sakai and Kikuchi, 2009). Besides, irresponsible teachers tend to refuse to encourage, care about, and communicate with students. Due to their irresponsibility and bad temper, they may seem unfriendly and unapproachable, thus hindering the teacher–student interaction and relationship. In this case, students are more likely to get demotivated (Unal and Yanpar Yelken, 2016). Moreover, some teachers depend too much on traditional teaching methods and fail to integrate modern technology into their English teaching. This will make students feel bored and demotivated (Song and Kim, 2017). Factors indirectly related to teachers include a teacher-centered class and the nature of class activities (Falout and Falout, 2005). In different countries, students have different degrees of enrollment pressure. To cultivate students’ ability to take tests, teachers must explain almost everything in a lesson, rarely providing students with the opportunity to present what they have learnt. This is especially true in grammar class. Consequently, students will participate less and less, making them feel alienated from the class and thus diminishing their motivation.

However, different research participants have their own understandings of teachers’ influence. Research with students as participants shows that teachers are the key factors resulting in students’ demotivation, while research with teachers as participants shows the opposite. For example, Chambers (1993) explored factors influencing students’ learning demotivation among 191 students in Grade 9 and seven teachers and found that students perceived as demotivated showed a lack of interest in what they were doing and were more likely to disrupt other students. They also showed no response to others’ help. Teachers believed that there were many reasons for students’ demotivation, including psychological, attitudinal, social, historical, and geographical factors. However, according to students, teachers were a major contributor to their demotivation. For example, teachers could not give correct instructions or their explanations were not clear enough to help students understand certain points. In addition, students would be criticized and scolded when they performed terribly. Consequently, there is a need to take types of participants into consideration when exploring students’ FLL demotivation.

What is worth our attention is that even among students, teachers’ influence varies with students of different levels. For example, using a questionnaire, Falout and Maruyama (2004) took 164 Japanese freshmen as participants and explored the influence of their experiences and negative emotions on their English learning demotivation. They divided these participants into low-proficient (LP) and high-proficient (HP) groups. The results showed that LP students tended to attribute their demotivation to internal factors, while HP students attributed it to external factors, especially teacher-related factors.

#### Curriculum-related factors

Curriculum-related factors mainly refer to features of the English language, boring learning contents, and tests (Falout and Falout, 2005). Learning English requires considerable recitation and memorization, of which many students are tired. What makes them even more exasperated is that they have to deal with boring learning materials and numerous tests. For example, Kikuchi (2009) explored 52 Japanese college students’ recollection of demotivators when they were learning English in high school. The results showed that college entrance exams and the nature of English courses and textbooks were among the five reasons for their demotivation. Some exams only focused on grammar and vocabulary. Students who could not perform well on these exams would be punished. Besides, there were many new words, idioms, sentences, and passages for them to remember. Learning English was not a pleasure for them but a burden. Therefore, these negative factors pulled students down and demotivated them.

#### Environment-related factors

According to the analysis of retrieved literature, environments that influence students’ FLL demotivation include the learning and social environments.

The learning environment mainly consists of school facilities (Song and Kim, 2017) and the classroom environment (Zhang et al., 2020). Poor school facilities will restrict teachers from adopting advanced teaching methods, thus affecting students’ interest in learning English and ultimately leading to their demotivation (Ushioda, 1998). For example, through a questionnaire, Sakai and Kikuchi (2009) explored English learning demotivators among 656 Japanese high school students. Through factor analysis, they identified lack of teaching equipment as a demotivator. There was no media equipment in some schools to enable teachers to select and use video and audio materials. Furthermore, some schools might have no access to the Internet to obtain the latest network resources. As a result, teachers had no choice but to use traditional teaching methods and could not enrich the variety of classroom activities. This prevented students from experiencing the integration of modern technology and English learning. In addition, the classroom environment, especially the teaching environment, will have an impact on students’ demotivation. For example, Unal and Yanpar Yelken (2016) employed a 20-item, four-dimensional scale with 454 pre-college students to explore their English learning demotivators. The results identified class environment as one of these demotivators, which was embodied by teachers who used minimal modern technology and relied heavily on traditional teaching methods. In other words, they could not integrate modern information technology into English teaching. In addition, teachers fail to check assigned tasks on time, reducing students’ enthusiasm to complete the tasks. Finally, teachers have a tendency to transfer knowledge without considering students’ actual needs and learning effects, thus making students feel ignored and demotivated.

Besides the learning environment, the social environment can have a terrible impact on students. This includes unsupportive family and the negative influence of important persons. First, whether parents support their children or not has a direct effect on the latter’s motivation. For example, with 6,301 primary school students from Grades 3 to 6 in South Korea, Kim (2011) explored how after-class English tutoring affected students’ motivation through a questionnaire. The results showed that whether parents provided financial support for students to attend after-class English tutoring affected students’ learning motivation, expectation, and satisfaction. Specifically, students with tutoring experience had higher learning motivation and satisfaction, while students without tutoring suffered from demotivation. In addition, some important people who are closely related to students may influence their demotivation, such as parents or classmates. For example, Hassaskhah et al. (2014) specified significant others as classmates, family members, and teachers. Explicitly speaking, parents’ unsupportive attitude to learning English, peers’ uncooperative participation in activities, and poor relationships among students would lead to learners’ demotivation. However, Hassaskhah et al. (2014) also regarded teachers as significant others. This might be because they regarded teachers as people with the same status as family members, classmates, and friends, and these were all influential individuals who greatly influenced students’ demotivation.

### The relationship between demotivation and other psychological factors

Students are complex and developmental individuals (Bronfenbrenner, 1986), and their demotivation is closely related to other psychological factors in the FLL process. In general, the level of demotivation is negatively correlated with positive psychological factors, such as increased self-confidence, and positively correlated with negative psychological factors, such as language anxiety.

Some researchers pay more attention to the effect of positive psychological factors on FLL demotivation. Kim’s team has conducted three studies on the relationship between FLL demotivation and other psychological factors among South Korean students in different grades. First, Shin and Kim (2017) administered a questionnaire to primary school students in Grade 6 to explore the effect of resilience on motivation and demotivation. The results showed that resilience positively affected students’ intrinsic motivation and ideal L2 self but had a negative effect on demotivation. Second, Kim et al. (2018) interviewed 23 students in different grades and nine teachers and identified that demotivators (e.g., teachers, after-school learning programs, and impractical EFL lessons) were stressors for students attempting to build resilience. Students’ resilience was significantly affected by social support, emotional regulation, clear learning goals, and tenacity in English learning. The stronger their resilience was, the weaker their demotivation became. Finally, Kim et al. (2019) investigated the influence of resilience on primary school students’ FLL motivation, demotivation, and language proficiency by administering a questionnaire to 367 Grade 6 students. Through factor analysis and structural equation modeling, the results indicated that the direct impact of resilience on English learning motivation was greater than its impact on demotivation. That is, building up resilience was more helpful in sustaining students’ motivation. In addition to Kim’s team, Pathan et al. (2020) explored potential language learning demotivators among Pakistani college students and their interactions with resilience and personality. They found that the demotivators were composed of external and internal factors, while resilience and personality were negatively correlated with these demotivators. This study suggested that English teachers adopt motivational teaching strategies to stimulate, enhance, and maintain language learners’ motivation and pay attention to the development of resilience, rigor, and openness to prevent students’ demotivation.

Other researchers have explored the relationship between negative psychological factors and demotivation. For example, Choi (2017) explored the relationship between anxiety and Japanese learning demotivation among Korean students. The results of this study showed that demotivation caused by peer stress was positively related to the anxiety associated with speaking Japanese and the worry about losing face. Besides, Zhang et al. (2020) investigated the effect of engagement and anxiety on L2 de/motivation among 591 undergraduate students. The results indicated that engagement could positively mediate the impacts of L2 de/motivation, while anxiety would hinder ELS achievement and lead to demotivation. This study represents the relationship between positive/negative psychological factors and demotivation clearly.

From what has been mentioned above, we know that researchers are interested in exploring FLL demotivators and have paid more attention to the relationship between demotivators and other psychological factors. By doing so, they have clearly identified demotivators and clarified their relationships with other psychological factors, both positive and negative.

### Methodological approaches In researching foreign language learning demotivation

Having conducted a content analysis of FLL demotivation studies, it is also necessary to gain a general understanding of how these studies were performed. Therefore, this section will briefly review the research design of the 28 selected studies on FLL demotivation in terms of their methods, participants, and instruments to determine how these studies were conducted.

First, according to Creswell and Creswell (2018), three research approaches are usually used in an empirical study—that is, quantitative, qualitative, and mixed-methods approaches. In this review, the selected 28 pieces of literature consist of quantitative studies (21 works), qualitative studies (4 works), and mixed-methods studies (3 works). As we can see, quantitative approaches dominate the demotivation research. Although it can be helpful to reflect the influence of demotivators in numerical terms, it is difficult to thoroughly describe the working mechanism of a single FLL demotivator, nor can this approach deeply explore the interaction between these demotivators and other psychological factors. Thus, researchers should take full advantage of qualitative and mixed methods to develop a comprehensive and in-depth description of demotivation. This will not only allow for identifying the factors that lead to students’ FLL demotivation but will also clarify the relationship between demotivators and other psychological factors.

Second, regarding participants, most of them are college students (16 works) and primary school students (7 works; e.g., Falout and Maruyama, 2004; Kim, 2011; Kim et al., 2019; Karaca and Inan, 2020). However, only five studies target high school students, which means that more attention should be paid to high school students’ FLL demotivation in order to stimulate their motivation.

Lastly, concerning research instruments, questionnaires, interviews, and surveys are the primary data collection tools. Researchers have also developed scales to explore FLL demotivators, such as DeMTB (demotivation test battery questionnaire; Hassaskhah et al., 2014) and EWDES (the English Writing Demotivation Scale; Karaca and Inan, 2020). However, these scales have not been used widely yet.

## Implications for future studies

Future research should strive to make breakthroughs by focusing on three aspects: expanding theoretical perspectives, including more research participants, and utilizing multiple research methods.

First, future research is suggested to expand the theoretical perspective. Dewaele et al. (2019) underlined that the application of interdisciplinary theories and methods contributed to the further development of the study of psychological factors in L2 acquisition. Therefore, in the process of exploring FLL demotivation, it is necessary to refer to relevant theories of other disciplines, especially general and educational psychology, such as Bronfenbrenner (1986) ecological systems theory. This theory places learners in a complex FLL ecosystem and studies the impact of the ecological environment on learners from different levels, such as macrosystem (e.g., national curriculum reform policies, social and cultural ideologies), exosystem (e.g., features of a foreign language curriculum), mesosystem (e.g., the relationship between teachers and learners’ parents and classmates), microsystem (e.g., the psychological change of peer classmates, teachers, parents, and learners), and chronosystem (e.g., events with an important impact). Especially, more attention should be diverted to the microsystem of classroom to arouse students’ language enjoyment and intrigue their learning motivation (Elahi Shirvan and Taherian, 2022). Using interdisciplinary theories and based on existing research findings on demotivation, a model of FLL demotivators can be constructed through comparisons with other disciplines.

Second, studies should include participants in different learning phases. Most research on FLL demotivation is aimed at college and primary school students, and there is a lack of in-depth exploration of middle school students. Middle school is an important transitional period for students from the basic FLL stage to the advanced learning stage in university. It is also an important period during which students’ psychology undergoes significant changes (Liu, 2010). Therefore, there is an urgent need to explore middle school students’ FLL demotivation. In addition, with the growth of students in middle school, the reasons for their FLL demotivation will become more complicated. Although some researchers have found that middle school students suffer from FLL demotivation (e.g., Sakai and Kikuchi, 2009; Choi, 2017; Gao et al., 2022), these studies have some limitations of small samples and elementary analysis. Therefore, investigating middle school students’ FLL demotivation will enrich the present findings and aid in understanding the effect that demotivators have on middle school students and their different reactions to demotivation. Exploring their demotivation will also be beneficial for exploiting their general sense of flow in FLL (Liu and Song, 2021) and stimulating their motivation.

Lastly, multiple research methods should be utilized to achieve triangulation. Future research should use not only quantitative but qualitative and mixed methods to conduct multi-angle explorations of FLL demotivation. FLL demotivation is a complex, multidimensional system, and it is the result of various factors at different levels working together on learners. Therefore, it is difficult to explore the interaction between various factors solely through quantitative research, let alone improve learners’ learning motivation. Therefore, future research can use various research methods, such as a qualitative method, mixed methods, and action research. For instance, a case study will ensure that demotivators and their interaction are thoroughly described, and action research will allow for exploring and practicing ways to improve learners’ motivation.

## Conclusion

Overall, we reviewed the definition of demotivation, the classification of demotivators, the relationships between these demotivators and other psychological factors, and methodological approaches. More importantly, we identified future research directions. Although research on FLL demotivation has increased significantly, scholars must explore it from different perspectives, within different groups, and *via* different research methods. Besides, future research should apply the fruits of positive psychology including positive emotions (e.g., foreign language enjoyment) to intrinsically motivating students to learn a foreign language (Jiang and Dewaele, 2019; Dewaele et al., 2022).

## Author contributions

LG: data collection and analysis, draft, and revision. HL: framework construction, data analysis, supervision, and revision. All authors contributed to the article and approved the submitted version.

## Funding

This paper was supported by the Project of Discipline Innovation and Advancement (PODIA) - Foreign Language Education Studies at Beijing Foreign Studies University (grant number: 2020SYLZDXM011) and the Project of Developmental Features of the Language Ability by Chinese Multilingual Second Language Learners-Youth Team Funding of Northeast Normal University, 2021 (grant number: 2021QT004).

## Conflict of interest

The authors declare that the research was conducted in the absence of any commercial or financial relationships that could be construed as a potential conflict of interest.

## Publisher’s note

All claims expressed in this article are solely those of the authors and do not necessarily represent those of their affiliated organizations, or those of the publisher, the editors and the reviewers. Any product that may be evaluated in this article, or claim that may be made by its manufacturer, is not guaranteed or endorsed by the publisher.
